# Duration of protection of CoronaVac plus heterologous BNT162b2 booster in the Omicron period in Brazil

**DOI:** 10.1038/s41467-022-31839-7

**Published:** 2022-07-18

**Authors:** Thiago Cerqueira-Silva, Vinicius de Araujo Oliveira, Enny S. Paixão, Juracy Bertoldo Júnior, Gerson O. Penna, Guilherme L. Werneck, Neil Pearce, Maurício L. Barreto, Viviane S. Boaventura, Manoel Barral-Netto

**Affiliations:** 1 LIB and LEITV Laboratories, Instituto Gonçalo Moniz, Fiocruz, Salvador, Bahia Brazil; 2 Universidade Federal da Bahia, Salvador, Bahia Brazil; 3 Center for Data and Knowledge Integration for Health (CIDACS), Instituto Gonçalo Moniz, Fiocruz, Salvador, Bahia Brazil; 4 London School of Hygiene and Tropical Medicine, London, WC1E 7HT UK; 5 Núcleo de Medicina Tropical, Universidade de Brasília. Escola Fiocruz de Governo, Fiocruz, DF Brazil; 6 Universidade do Estado do Rio de Janeiro, Rio de Janeiro, Brazil; 7 Universidade Federal do Rio de Janeiro, Rio de Janeiro, Brazil

**Keywords:** Epidemiology, Outcomes research, Vaccines, SARS-CoV-2

## Abstract

To date, no information has been published on the effectiveness of inactivated whole-virus COVID-19 vaccines plus heterologous booster against symptomatic infection and severe outcomes (hospitalization or death) during the dominance of the SARS-CoV-2 Omicron variant period. We evaluated the vaccine effectiveness (VE) of CoronaVac plus BNT162b2 booster during the period of dominance of the Omicron variant in Brazil (January to April 2022). Using a test-negative design, we analysed data for 2,471,576 individuals tested during the Omicron variant’s dominant period using a nationally linked database from Brazil. Compared to unvaccinated, vaccinees maintained protection against severe outcomes, with an estimated VE of 84.1% (95% CI:83.2–84.9) at more than 120 days after BNT162b2 booster. Furthermore, while we detected a high level of protection against severe outcomes for individuals up to 79 years old, waning was observed for individuals aged ≥80 years, with VE decreasing from 81.3% (95% CI:77.9–84.2) at 31–60 days to 72.9% (95% CI:70.6–75.1) at 120 days or more after the booster dose. However, no significant protection against symptomatic infection was observed at this time period. In conclusion, except for individuals aged ≥80 years, CoronaVac plus a BNT162b2 booster dose offered high and durable protection against severe outcomes due to Omicron.

## Abstract (web summary)

Primary CoronaVac vaccination followed by a BNT162b2 booster dose confers protection against some SARS-CoV-2 variants but its effectiveness against Omicron is unknown. Here, the authors show that this combination confers a high level of protection against severe outcomes for up to 120 days, with evidence of waning for those aged 80 or older.

## Introduction

SARS-CoV-2 variants and progressive waning impacted the effectiveness of all COVID-19 vaccines^1,2^. The administration of a booster dose 3–6 months after the primary series increased protection against symptomatic infection and hospitalization^2–4^. The Omicron variant affected the duration of this protection as several reports have suggested a significant decrease in protection against infection; this has resulted in the offer of a second booster by some countries^5,6^. However, there is little published information about the effectiveness of the booster doses against severe outcomes caused by the Omicron variant^7,8^. Furthermore, none of these studies has included CoronaVac vaccinees as the primary series, followed by a BNT162b2 booster dose.

Inactivated vaccines have been the most used worldwide, with more than (4.7 billion-40% of all doses) manufactured until January 2022^9^. Coronavac has been approved for use in 54 Low and Middle-income countries^10^. However, the number of studies investigating the effectiveness of CoronaVac has been far lower than for other vaccines. Studies of the effectiveness of the booster dose in different age groups after the emergence of Omicron are needed to provide evidence to guide the eventual indication of a second booster dose.

The older people deserve special attention regarding protection against Omicron, considering their increased risk of severe COVID-19 and the most extended interval between the booster administration and the Omicron dominance compared to other age groups. The older people already showed lower protection against Gamma and Delta variants after the second dose than younger people^11^. In Brazil, most older people individuals have received two doses of CoronaVac and BNT162b2 booster^2,11^. Thus, data on VE during the Omicron period will provide evidence for orienting the further steps of vaccination rollout in the countries using Coronavac.

Here, we used a nationwide linked database to evaluate the effectiveness of a heterologous BNT162b2 booster in individuals vaccinated with CoronaVac during the Omicron period in Brazil. We evaluated protection against symptomatic infection and severe outcomes (hospitalization and death) by age group, in vaccinees that received a booster dose compared to either unvaccinated or individuals who received only two CoronaVac doses.

## Results

**Fig. 1:**
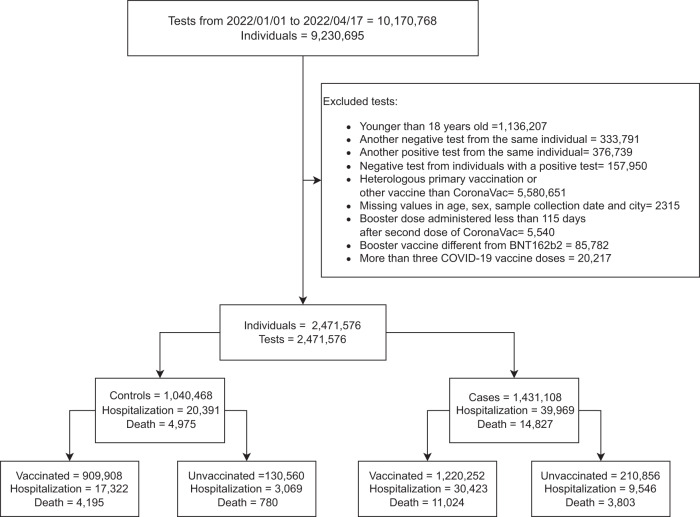
Flowchart of the study population from surveillance databases and selection of cases and controls. Only lateral flow or RT–PCR tests with the sample collected within 10 of symptom onset were considered eligible. Sensitivity analysis was performed by removing unvaccinated individuals (cases and controls).

**Fig. 2:**
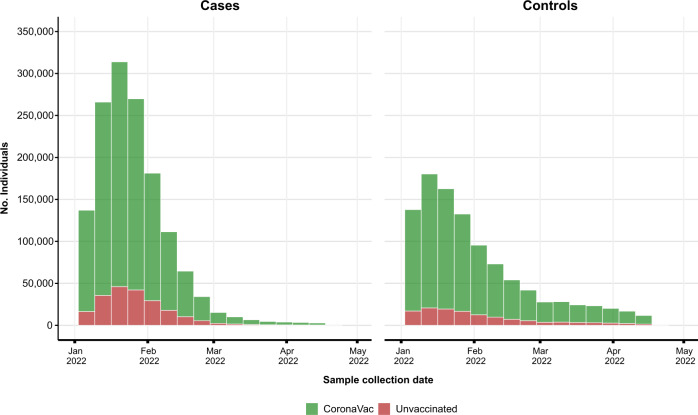
Number of cases and controls, by week, during the study period, stratified by vaccination status. CoronaVac vaccinees refer to individuals with at least one dose of CoronaVac.

From January 01, 2022, to April 17, 2022, the period of predominant circulation of Omicron in Brazil (Supplementary Fig. 1). 9,230,695 symptomatic individuals were tested in this period, and 2,471,576 individuals were selected (Fig. 1). Among them, 2,130,160 individuals were vaccinated with at least one dose of CoronaVac, and 341,416 were unvaccinated individuals (Table 1). The majority of tests were positive, with 1,220,252 (57.3%) tests of the vaccinated group and 210,856 (61.8%) of the unvaccinated group (Table 1/Fig. 2). The vaccinated group had more women than the unvaccinated group (60.2% vs 48.3%) (Supplementary Table 1). A total of 852,911 (40.0%) individuals received a BNT162b2 booster dose (Table 1). Additional information is provided in Supplementary Tables 1–3.

**Table 1:** Clinical and sociodemographic characteristics of individuals tested by SARS-CoV-2 RT–PCR or Rapid Antigen.

Characteristic	Positive, *N* = 1,431,108	Negative, *N* = 1,040,468
Age - years	36 (27–55)	36 (27–56)
Age group - years		
18–59	1,108,629 (77.5)	799,283 (76.8)
60–79	273,532 (19.1)	207,988 (20.0)
≥ 80	48,947 (3.4)	33,197 (3.2)
Sex-Female	831,102 (58.1)	615,083 (59.1)
Residence in capital state	306,844 (21.4)	256,961 (24.7)
Type of test		
Lateral-flow	1,122,581 (78.4)	867,535 (83.4)
RT-PCR	308,527 (21.6)	172,933 (16.6)
Municipality Deprivation Index		
1 (Least)	470,517 (32.9)	365,662 (35.1)
2	291,321 (20.4)	204,225 (19.6)
3	271,329 (19.0)	191,162 (18.4)
4	242,583 (17.0)	158,513 (15.2)
5 (Most)	154,969 (10.8)	120,687 (11.6)
(Missing)	389 (0.0)	219 (0.0)
Diabetes Mellitus	45,604 (3.2)	35,257 (3.4)
Obesity	12,804 (0.9)	9216 (0.9)
Immunosuppression	7973 (0.6)	5956 (0.6)
Chronic respiratory disease	33,874 (2.4)	33,292 (3.2)
Cardiac disease	76,449 (5.3)	59,952 (5.8)
Chronic Kidney Disease	5614 (0.4)	3481 (0.3)
No. comorbidities		
0	1,291,056 (90.2)	927,103 (89.1)
1	105,233 (7.4)	85,396 (8.2)
2	28,411 (2.0)	22,962 (2.2)
≥3	6408 (0.4)	5007 (0.5)
Previous SARS-CoV-2 infection		
No	1,330,372 (93.0)	928,025 (89.2)
3–6 months ago	5517 (0.4)	9808 (0.9)
>6 months ago	95,219 (6.7)	102,635 (9.9)
Vaccination Status		
Unvaccinated	210,856 (14.7)	130,560 (12.5)
First dose CoronaVac	84,989 (5.9)	65,428 (6.3)
Second dose CoronaVac	687,233 (48.1)	439,599 (42.3)
Booster dose BNT162b2	448,030 (31.3)	404,881 (38.9)
Hospitalization	39,969 (2.8)	20,391 (2.0)
Death	14,827 (1.0)	4975 (0.5)
Severe outcome	42,340 (3.0)	21,393 (2.1)

*n* (%); Median (IQR).

### Main analysis

**Fig. 3:**
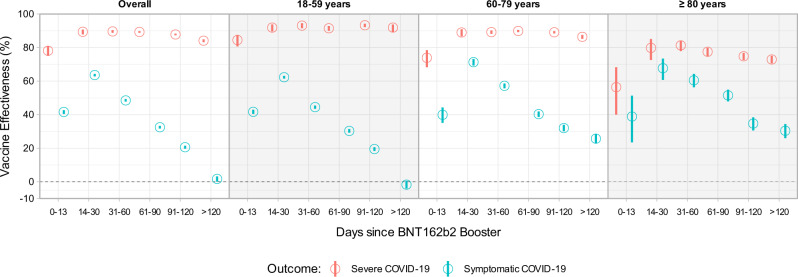
Vaccine Effectiveness against symptomatic and Severe COVID-19. According to days after booster dose during the Omicron dominance period, stratified by age group. Point estimates are adjusted vaccine effectiveness (1- adjusted odds ratio), with error bars indicating the corresponding 95% Wald’s C.I. Blue represents adjusted VE against symptomatic infection, and red adjusted VE against severe outcomes. All models the comparison group is unvaccinated.

We first estimated the vaccine effectiveness (VE) of the Coronavac vaccine plus BNT162b2 booster using unvaccinated individuals as the comparison group. Protection against symptomatic infection by Omicron decreased substantially from 63.6 (95% CI: 62.8 to 64.3%) at 14–30 days to 1.7% (95% CI: 0.1 to 3.2) at 120 or more days after the booster dose. VE peaked at around 60% in all age groups and decreased to equal to or lower than 30% after 120 days or more. VE against symptomatic infection was lowest in the 18–59 age group, reaching −1.7% (95% CI: −4.0 to 0.5) after 120 days of more (Fig. 3/Supplementary Table 4).

Estimated VE against severe outcomes (hospitalization or death) was 89.4% (95% CI: 87.8 to 90.7) at 14–30 days, waning to 84.1% (95% CI: 83.2 to 84.9) at 120 days or more after the booster dose. It varied according to the age group; among younger individuals (18–59), the VE remained highly effective over time, peaking at 31–60 days (93.1%, 95% CI: 91.5 to 94.5) and maintaining a similar level at 120 or more days (91.9%, 95% CI: 89.4 to 93.7) after the booster. For the subgroup of 60–79, all estimates after 14 days post booster dose remained higher than 85%, reaching a peak at 61–90 days (89.9%, 95% CI: 89.2to 90.5). Among individuals aged 80 years or older, the VE peak occurred at 31–60 days (81.3% (95% CI: 77.9 to 84.2) waning to 72.9% (70.6 to 75.1) more than 120 days after the booster dose (Fig. 3/Supplementary Table 4).

Given the rise of incidental COVID-19 hospitalizations^12^, and concerns about possibly biased VE estimates in analyzing the composed outcome, we also evaluated the VE against death alone. The overall protection remained close to 90% at 14 to 120 days, decreasing to 87.0% (95% CI: 85.9 to 88.0) after 120 or more days after a booster dose. After 120 days post booster, the VE among the age-groups was 93.8% (95% CI: 88.8 to 96.6) for 18–59 years, 89.9 (95% CI: 88.4–91.2) for 60–79 years, and 80.2% (78.0 to 82.3) for individuals 80 years or older (Supplementary Table 5).

### Sensitivity analysis

We also conducted a sensitivity analysis using as the reference group individuals with more than 180 days of the second doses of CoronaVac but without the booster dose to estimate the additional protection conferred by the booster dose. This aims to remove potential bias due to different behaviors between vaccinated and unvaccinated individuals^13^. A similar pattern of the waning in protection against symptomatic infection and maintenance of protection against severe outcomes were observed overall and by age groups (Supplementary Fig. 2 and Supplementary Table 6). For individuals aged 80 years or older, a booster dose with BNT162b2 increased the protection against severe outcomes by 65.3 % (95% CI: 53.0 to 74.3) at 14–30 days and 52.8% (95% CI: 49.2 to 56.1) more than 120 days after booster compared to those with only two doses of CoronaVac after more than 6 months from the last dose.

To address possible differences in accuracy of diagnostic tests for COVID-19^14^, we stratified the analysis of VE by type of test. Results were similar to the main analysis (Supplementary Tables 7 and 8).

## Discussion

During the period that the Omicron variant dominance, individuals CoronaVac-vaccinees and boosted with BNT162b2 exhibited highly effective protection against severe forms of COVID-19, but a fast waning against symptomatic infection across all age groups in Brazil.

Our findings of VE against both symptomatic infection and severe outcomes with the Omicron variant obtained with two Coronavac doses plus BNT162b2 booster are consistent with results of studies investigating protection of primary series of ChAdOx1 plus booster with the mRNA vaccine^7,8,15^ or three doses of BNT162b2 vaccine^8^. In Finland, among individuals aged 70 years or older VE against hospitalization in the Omicron period reached 90% after 2 months of the booster dose for individuals vaccinated with a primary series of BNT162b2 or ChAdox1 plus a BNT162 booster^15^. Our study found a VE higher than 85% at least for 4 months in individuals between 60 and 79 years old. Regarding waning against Omicron-related symptomatic infection, we report the peak of protection in the first month after the BNT162b2 booster, followed by a sharp decrease in VE. These results are similar to those reported for individuals that received primary series with ChAdOx1, mRNA-1273, BNT162b2, or Ad26.cov2.S^16,17^. Together, these findings suggest that a booster dose of mRNA vaccines after a primary series of vaccines from different platforms maintains adequate protection against severe outcomes despite failing to protect against Omicron symptomatic infection. Continuous monitoring should be performed to detect early warnings of waning protection against hospitalization or death.

We observed a lower VE against symptomatic infection for individuals aged up to 59 years old compared to older individuals. We hypothesize this can be related to changes in behavior post-booster-vaccination, lowering the risk perception of getting COVID-19, and adherence to personal preventive measures^18,19^, mainly in the younger group^20,21^, increasing the risk of infection by the Omicron variant.

Subgroup analysis by age showed a significant and rapid waning of protection for the older people. The impact of immune senescence has already been reported after the primary series. The benefit of a second booster should take into account that there is reasonable protection against severe outcomes, and eventual protection against symptomatic infection may be ephemeral^5^.

A critical strength of this study is that consistent results were obtained for the two different reference groups used: individuals unvaccinated or that received only two CoronaVac doses more than 6 months before the RT-PCR or rapid antigen test. Both analyses observed similar patterns over time – protection maintenance against severe outcomes and fast waning against symptomatic infection. However, this study has some limitations. First, the rapid and expressive spread of the Omicron variant was associated with a high rate of positivity in COVID-19 tests in Brazil, suggesting that many positive cases were not tested. Second, the majority of the performed tests during the study period were lateral-flow ones, which may occur in misclassifying cases as controls. The individuals aged ≥ 80 years were more tested with RT-PCR (29%) than the younger ones (19% each), which may reflect the more restricted use of RT-PCR tests at hospitals combined with the increased risk of severity in this older group. However, the analysis stratifying by type of test provided small differences in the points estimates with similar trends. Although the test-negative design is considered the best model to avoid bias in the access to tests and healthcare-seeking behavior, individuals that got tested may differ from those that were not tested, potentially impacting the external validity of the results.

In conclusion, two doses of CoronaVac plus a BNT162b2 booster led to protection against severe outcomes, being robust and stable for at least 4 months for most age groups. In contrast, there is a lack of consistent protection against Omicron symptomatic infection by vaccines using different platforms and heterologous boosters with the current vaccines. The perspective is for SARS-CoV-2 to continue circulating worldwide, even in places with high vaccine coverage. Therefore, the possible emergence of new variants of concern highlights the necessity to develop new vaccines that, besides severe disease, also prevent infection, providing durable protection against infection and disease.

## Methods

### Study design and data sources

We used a test negative design, a study design widely used for evaluating vaccine effectiveness (VE) in Influenza and SARS-CoV-2^22^. We evaluated the VE of CoronaVac plus a booster dose of BNT162b2 on symptomatic individuals tested with RT-PCR or Lateral-flow tests.

From January 2022 onwards, the Omicron variant was dominant in Brazil. (Supplementary Fig. 1). The Brazilian Ministry of Health started recommending a booster dose on September 15, 2021, initially 6 months after the second CoronaVac dose, reduced to 4 months on December 20, 2021, primarily with BNT162b2.

We analyzed a deterministically linked dataset comprised of three databases (Supplementary Fig. 3): the Programa Nacional de Imunizações (PNI); the e-SUS Notifica; and the Sistema de Informação da Vigilância Epidemiológica da Gripe (SIVEP-Gripe), described previously^11,23^. All data were pseudo-anonymized with a common unique identifier provided by the Brazilian Ministry of Health. The research protocol was approved by the Brazilian National Commission in Research Ethics (CONEP) (approval number 4.921.308).

All individuals aged 18 years or older who reported COVID-19-like symptoms and were tested for SARS-CoV-2 between January 01, 2022, and April17, 2022 were eligible for the study. Cases and controls were defined as individuals with RT-PCR/Lateral-flow test positive or negative, respectively. Cases of COVID-19 hospitalization were defined by a positive SARS-CoV-2 test if the positive specimen was collected up to 14 days before or 3 days after the hospital admission, and cases of COVID-19 death were defined by death occurring within 28 days of the sample collection date. The same set of controls was used for all analyses. For the analysis of severe outcomes, the mild cases were excluded and for the death outcome, mild and hospitalized cases were excluded. The exclusion criteria for tests were: (i) tests from individuals younger than 18 years; (ii) tests from individuals who received a different vaccine for the second dose from the first; (iii) tests from individuals whose time interval between the first and second doses was less than 14 days; (iv) tests from individuals with less than 115 days between the second and booster dose (outside the interval between doses officially recommended in Brazil); (v) tests with missing information of age, sex, city of residence or sample collection date; (vi) negative tests from individuals with a positive test; (vii) more than three doses of COVID-19 vaccines. Only the first positive test during the study period was included for each case, and for controls, only the first negative test was included.

### Statistical analysis

The odds ratio (OR) comparing odds of vaccination between cases and controls, and its associated 95% confidence interval (CI) were derived using generalized additive logistic regression, adjusting for potential confounders: age, sex, temporal trends, state of residence, previous infection, municipality deprivation index, and comorbidities. The temporal trend was estimated using the time elapsed, in days, between the study start and the date of symptoms onset. Temporal trends and age were modeled as cubic regression spline smooth functions. The comorbidities were cardiac disease, diabetes mellitus, obesity, immunosuppression, chronic respiratory disease, and chronic kidney disease. The VE was estimated as 1-OR and expressed as a percentage. Vaccination status, according to the status at the time of specimen test collection, were classified as unvaccinated and grouped in periods (days) after each dose: first dose (0–13, ≥14), second dose (0–13,14–180, >180) and a booster dose (0–13, 14–30, 31–60, 61–90, 91–120, >120). Analyses were also performed stratified by age groups (18–59, 60–79, and ≥80 years). As a sensitivity analyses, we performed stratified analysis by type of test and we also compared individuals with a booster dose against individuals with the second dose over 180 days. All data processing and analyses were performed in R (version 4.1.2)^24^, using the following packages: tidyverse^25^ and mgcv^26^.

### Reporting summary

Further information on research design is available in the Nature Research Reporting Summary linked to this article.

## Supplementary information

Reporting Summary

Supplementary Information

## Data Availability

One of the study coordinators (M.B.-N.) signed a term of responsibility on using each database made available by the Ministry of Health (MoH). Each member of the research team signed a term of confidentiality before accessing the data. Data was manipulated in a secure computing environment, ensuring protection against data leakage. The Brazilian National Commission in Research Ethics approved the research protocol (CONEP approval number 4.921.308). Our agreement with MoH for accessing the databases patently denies authorization of access to a third party. Any information for assessing the databases must be addressed to the Brazilian MoH at https://datasus.saude.gov.br/, and requests can be addressed to datasus@saude.gov.br. Herein we used anonymized secondary data following the Brazilian Personal Data Protection General Law (LGPD), but it is vulnerable to re-identification by third parties, as they contain dates of relevant health events regarding the same person. To protect the research participants’ privacy, the approved Research Protocol (CONEP approval number 4.921.308) authorizes only the dissemination of aggregated data, such as the data presented here.

## Code availability

The code used for this study is deposited in the git repository: https://github.com/csthiago/duration_heterologous_coronavac^27^.

## References

[1] Nordström P, Ballin M, Nordström A (2022). Risk of infection, hospitalisation, and death up to 9 months after a second dose of COVID-19 vaccine: a retrospective, total population cohort study in Sweden. Lancet.

[2] Cerqueira-Silva, T. et al. Vaccine effectiveness of heterologous CoronaVac plus BNT162b2 in Brazil. *Nat. Med*. 1–6 10.1038/s41591-022-01701-w. (2022). 10.1038/s41591-022-01701-w PMC9018414 35140406

[3] Bar-On YM (2021). Protection of BNT162b2 Vaccine Booster against Covid-19 in Israel. N. Engl. J. Med..

[4] Andrews, N. et al. Effectiveness of COVID-19 booster vaccines against COVID-19-related symptoms, hospitalization and death in England. *Nat. Med*. 1–7 10.1038/s41591-022-01699-1. (2022). 10.1038/s41591-022-01699-1 PMC9018410 35045566

[5] Regev-Yochay G (2022). Efficacy of a fourth dose of Covid-19 mRNA vaccine against omicron. N. Engl. J. Med..

[6] Smith, J. & Shin, H. South Korea to start giving fourth doses of COVID vaccine. *Reuters* (2022).

[7] Thompson, M. G. Effectiveness of a Third Dose of mRNA Vaccines Against COVID-19–Associated Emergency Department and Urgent Care Encounters and Hospitalizations Among Adults During Periods of Delta and Omicron Variant Predominance — VISION Network, 10 States, August 2021–January 2022. *MMWR Morb. Mortal. Wkly. Rep*. **71** (2022). 10.15585/mmwr.mm7104e3 PMC9351525 35085224

[8] Tartof, S. Y. et al. Durability of BNT162b2 vaccine against hospital and emergency department admissions due to the omicron and delta variants in a large health system in the USA: a test-negative case-control study. *Lancet Respir. Med*. S2213-2600(22)00101–1 10.1016/S2213-2600(22)00101-1. (2022). 10.1016/S2213-2600(22)00101-1 PMC9033225 35468336

[9] COVID 19 Vaccine production to January 31st 2022 - Global Commission for Post-Pandemic Policy. https://globalcommissionforpostpandemicpolicy.org/covid-19-vaccine-production-to-january-31st-2022.

[10] Sinovac: CoronaVac – COVID19 Vaccine Tracker. https://covid19.trackvaccines.org/vaccines/7/.

[11] Cerqueira-Silva T (2022). Influence of age on the effectiveness and duration of protection of Vaxzevria and CoronaVac vaccines: A population-based study. Lancet Reg. Health - Am..

[12] Bouzid, D. et al. Comparison of patients infected with delta versus omicron COVID-19 variants presenting to paris emergency departments. *Ann. Intern. Med*. 10.7326/M22-0308. (2022). 10.7326/P22-0005 PMC8949738 35286145

[13] Fridman A, Gershon R, Gneezy A (2021). COVID-19 and vaccine hesitancy: A longitudinal study. PLOS ONE.

[14] Peto, T. et al. COVID-19: Rapid antigen detection for SARS-CoV-2 by lateral flow assay: A national systematic evaluation of sensitivity and specificity for mass-testing. *eClinicalMedicine* **36**, (2021). 10.1016/j.eclinm.2021.100924 PMC8164528 34101770

[15] Baum, U. et al. High vaccine effectiveness against severe Covid-19 in the elderly in Finland before and after the emergence of Omicron. *medRxiv* 2022.03.11.22272140 10.1101/2022.03.11.22272140. (2022). 10.1186/s12879-022-07814-4 PMC9636823 36335289

[16] Monge, S. *et al*. The Effectiveness of mRNA Vaccine Boosters for Laboratory-Confirmed COVID-19 During a Period of Predominance of the Omicron Variant of SARS-CoV-2. *SSRN*10.2139/ssrn.4035396. (2022).

[17] Andrews N (2022). Covid-19 vaccine effectiveness against the omicron (B.1.1.529) variant. N. Engl. J. Med..

[18] Corea F, Folcarelli L, Napoli A, del Giudice GM, Angelillo IF (2022). The impact of COVID-19 vaccination in changing the adherence to preventive measures: evidence from Italy. Vaccines.

[19] Zhang N (2021). Weakening personal protective behavior by Chinese university students after COVID-19 vaccination. Build. Environ..

[20] Hutchins HJ (2020). COVID-19 mitigation behaviors by age group - United States, April-June 2020. MMWR Morb. Mortal. Wkly. Rep..

[21] Monod M (2021). Age groups that sustain resurging COVID-19 epidemics in the United States. Science.

[22] Chua H (2020). The use of test-negative controls to monitor vaccine effectiveness: a systematic review of methodology. Epidemiol. Camb. Mass.

[23] Katikireddi SV (2022). Two-dose ChAdOx1 nCoV-19 vaccine protection against COVID-19 hospital admissions and deaths over time: a retrospective, population-based cohort study in Scotland and Brazil. Lancet.

[24] R Core Team. *R: A Language and Environment for Statistical Computing*. (R Foundation for Statistical Computing, 2021).

[25] Wickham H (2019). Welcome to the Tidyverse. J. Open Source Softw..

[26] Wood SN (2004). Stable and efficient multiple smoothing parameter estimation for generalized additive models. J. Am. Stat. Assoc..

[27] Thiago *et al*. *Duration of protection of CoronaVac plus heterologous BNT162b2 booster in the Omicron period in Brazil*. (Zenodo, 2022). 10.5281/zenodo.6672955. 10.1038/s41467-022-31839-7 PMC9289933 35851597

