# Supplementary material for: Duration of protection of CoronaVac plus heterologous BNT162b2 booster in the Omicron period in Brazil
Source: Nat Commun. 2022 Jul 18;13:4154. doi: 10.1038/s41467-022-31839-7 (PMC9289933; doi:10.1038/s41467-022-31839-7)
Supplement: Supplementary file 2 — Supplementary Information [file 41467_2022_31839_MOESM2_ESM.pdf]

Contents

Supplementary Table 1: Clinical and sociodemographic characteristics of individuals tested for SARS-CoV-2 by vaccination status 2

Supplementary Table 2: Vaccination status of individuals tested for SARS-CoV-2 in Brazil........ 3

Supplementary Table 3: Percentage of type of test and length of follow-up in the group >120 days past booster dose, stratified by age group. 4

Supplementary Table 4: Vaccine effectiveness [%-(95% CI)] against symptomatic infection and severe outcomes associated with COVID-19 during the Omicron dominance period, stratified by age group. 5

Supplementary Table 5: Vaccine effectiveness [%-(95% CI)] against death associated with COVID-19 during the Omicron dominance period, stratified by age group 6

Supplementary Table 6: Adjusted odds ratio (95% CI) comparing odds of a booster dose in cases and controls, reference group individuals past 180 days of the second dose without a booster. Models for symptomatic and severe COVID-19. Odds ratio according to days past booster dose during the Omicron dominance period, stratified by age group. 7

Supplementary Table 7: Vaccine effectiveness [%-(95% CI)] against symptomatic SARS-CoV-2 infection during the Omicron dominance period, stratified by type of test and age group. 9

Supplementary Table 8: Vaccine effectiveness against [%-(95% CI)] severe outcomes associated with COVID-19 during the Omicron dominance period, stratified by type of test and age group. 10

Supplementary Figure 1: Monthly prevalence of SARS-CoV-2 variants in Brazil among genotyped isolates in the GISAID (global initiative on sharing avian influenza data). 11

Supplementary Figure 2: Adjusted odds ratio comparing odds of a booster dose in cases and controls. Models for symptomatic and severe COVID-19. Odds ratio according to days post booster dose during the Omicron dominance period, stratified by age group. Circles are adjusted odds ratio, with error bars indicating the corresponding 95% Wald's C.I. 12

Supplementary figure 3- Diagram showing the data linkage process 13

The RECORD statement – checklist of items, extended from the STROBE statement, that should be reported in observational studies using routinely collected health data. 14

Supplementary Table 1: Clinical and sociodemographic characteristics of individuals tested for SARS-CoV-2 by vaccination status

Characteristic	CoronaVac*, N = 2,130,160	Unvaccinated, N = 341,416
Individuals	2,130,160 (100.0)	341,416 (100.0)
Age - years, median (IQR)	36 (27 – 58)	37 (27 – 49)
Age group - years		
18-59	1,607,137 (75.4)	300,775 (88.1)
60-79	449,145 (21.1)	32,375 (9.5)
≥ 80	73,878 (3.5)	8,266 (2.4)
Sex-Female	997,773 (60.0)	208,161 (51.3)
Residence in capital	500,461 (23.5)	63,344 (18.6)
Test type		
Lateral-flow	1,710,619 (80.3)	279,497 (81.9)
RT-PCR	419,541 (19.7)	61,919 (18.1)
Deprivation Index		
1 (least)	729,774 (34.3)	106,405 (31.2)
2	436,192 (20.5)	59,354 (17.4)
3	400,655 (18.8)	61,836 (18.1)
4	337,182 (15.8)	63,914 (18.7)
5 (most)	225,901 (10.6)	49,755 (14.6)
(Missing)	456 (0.0)	152 (0.0)
Diabetes	73,139 (3.4)	7,722 (2.3)
Obesity	19,237 (0.9)	2,783 (0.8)
Immunosuppression	11,613 (0.5)	2,316 (0.7)
Chronic respiratory disease	59,211 (2.8)	7,955 (2.3)
Cardiac disease	122,365 (5.7)	14,036 (4.1)
Chronic Kidney Disease	7,886 (0.4)	1,209 (0.4)
No. comorbidities		
0	1,905,070 (89.4)	313,089 (91.7)
1	168,642 (7.9)	21,987 (6.4)
2	46,190 (2.2)	5,183 (1.5)
3+	10,258 (0.5)	1,157 (0.3)
Previous SARS-CoV-2 infection		
No	1,941,263 (91.1)	317,134 (92.9)
3-6 months ago	13,128 (0.6)	2,197 (0.6)
>6 months ago	175,769 (8.3)	22,085 (6.5)
Test Result		
Positive	1,220,252 (57.3)	210,856 (61.8)
Negative	909,908 (42.7)	130,560 (38.2)
Hospitalization	47,745 (2.2)	12,615 (3.7)
Death	15,219 (0.7)	4,583 (1.3)
Severe outcome	50,263 (2.4)	13,470 (3.9)

n (%); Median (IQR)

* Individuals with at least one dose of CoronaVac vaccine.

Supplementary Table 2: Vaccination status of individuals tested for SARS-CoV-2 in Brazil

	18 - 59			60-79			≥ 80 years			Overall		
	Cases		Controls	Cases		Controls	Cases		Controls	Cases		Controls
	Symptomatic	Severe		Symptomatic	Severe		Symptomatic	Severe		Symptomatic	Severe	
**Vaccination Status**												
Unvaccinated	179,890 (59.8)	3,834 (1.3)	117,051 (38.9)	17,848 (55.1)	3,682 (11.4)	10,845 (33.5)	2,921 (35.3)	2,681 (32.4)	2,664 (32.2)	200,659 (58.8)	10,197 (3.0)	130,560 (38.2)
1st dose 0-13	1,354 (51.1)	24 (0.9)	1,273 (48.0)	42 (44.7)	11 (11.7)	41 (43.6)	5 (27.8)	6 (33.3)	7 (38.9)	1,401 (50.7)	41 (1.5)	1,321 (47.8)
1st dose ≥14 days	77,222 (55.9)	635 (0.5)	60,215 (43.6)	3,867 (50.7)	625 (8.2)	3,139 (41.1)	683 (35.0)	515 (26.4)	753 (38.6)	81,772 (55.4)	1,775 (1.2)	64,107 (43.4)
2nd dose 0-13	2,730 (52.4)	18 (0.3)	2,466 (47.3)	49 (62.8)	3 (3.8)	26 (33.3)	2 (28.6)	3 (42.9)	2 (28.6)	2,781 (52.5)	24 (0.5)	2,494 (47.1)
2nd dose 14-180 days	496,839 (61.6)	2,026 (0.3)	307,892 (38.2)	2,920 (56.4)	329 (6.4)	1,925 (37.2)	372 (41.5)	220 (24.5)	305 (34.0)	500,131 (61.5)	2,575 (0.3)	310,122 (38.2)
2nd dose >180 days	112,704 (56.7)	897 (0.5)	85,129 (42.8)	49,960 (54.3)	6,902 (7.5)	35,222 (38.2)	6,839 (38.2)	4,420 (24.7)	6,632 (37.1)	169,503 (54.9)	12,219 (4.0)	126,983 (41.1)
Booster with BNT162b2												
0-13	22,869 (50.7)	88 (0.2)	22,174 (49.1)	1,719 (53.4)	159 (4.9)	1,340 (41.6)	144 (38.8)	83 (22.4)	144 (38.8)	24,732 (50.8)	330 (0.7)	23,658 (48.6)
14-30	15,635 (39.0)	61 (0.2)	24,367 (60.8)	1,535 (34.5)	148 (3.3)	2,768 (62.2)	136 (26.6)	67 (13.1)	309 (60.4)	17,306 (38.4)	276 (0.6)	27,444 (61.0)
31-60	30,106 (44.4)	92 (0.1)	37,588 (55.5)	14,934 (45.2)	760 (2.3)	17,378 (52.5)	782 (33.7)	245 (10.6)	1,293 (55.7)	45,822 (44.4)	1,097 (1.1)	56,259 (54.5)
61-90	48,422 (49.3)	153 (0.2)	49,598 (50.5)	65,200 (54.7)	2,544 (2.1)	51,450 (43.2)	2,776 (39.5)	810 (11.5)	3,440 (49.0)	116,398 (51.9)	3,507 (1.6)	104,488 (46.6)
91-120	91,482 (58.9)	204 (0.1)	63,595 (41.0)	74,293 (56.9)	3,482 (2.7)	52,857 (40.5)	10,420 (49.0)	2,465 (11.6)	8,372 (39.4)	176,195 (57.4)	6,151 (2.0)	124,824 (40.6)
>120	21,280 (43.2)	64 (0.1)	27,935 (56.7)	21,155 (39.5)	1,365 (2.6)	30,997 (57.9)	9,633 (44.5)	2,719 (12.6)	9,276 (42.9)	52,068 (41.8)	4,148 (3.3)	68,208 (54.8)

N (row % by subgroup)

Supplementary Table 3: Percentage of type of test and length of follow-up in the group >120 days past booster dose, stratified by age group.

Characteristic	18-59 years<br>N = 1,907,912	60-79 years<br>N = 481,520	≥80 years<br>N = 82,144
Type of test			
Lateral-flow	1,539,702 (80.7)	391,877 (81.4)	58,537 (71.3)
RT-PCR	368,210 (19.3)	89,643 (18.6)	23,607 (28.7)
Days since booster in the group >120 days, median - IQR	133 (126 – 148)	136 (127 – 149)	136 (127 – 150)

[1] n (%) | IQR=Interquartile range

Supplementary Table 4: Vaccine effectiveness [%-(95% CI)] against symptomatic infection and severe outcomes associated with COVID-19 during the Omicron dominance period, stratified by age group.

Vaccination Status	Symptomatic Infection				Severe Outcomes			
	Overall	18-59 years	60-79 years	≥ 80 years	Overall	18-59 years	60-79 years	≥ 80 years
First dose								
≥ 14	15.5 (14.4 — 16.6)	14.1 (12.9 — 15.3)	28.3 (24.3 – 32.0)	24.7 (16.0 — 32.5)	51.8 (48.7 — 54.8)	53.3 (48.9 — 57.4)	55.3 (50.1 — 60.0)	32.9 (22.5 — 42.0)
Second dose								
14-180	-0.7 (-1.6 — 0.2)	-1.3 (-2.6 — -0.1)	17.8 (12.4 — 22.8)	15.1 (0.9 — 27.3)	64.5 (62.6 — 66.3)	69.2 (67.3 — 71.1)	50.2 (42.5 — 56.9)	44.3 (31.2 — 54.9)
> 180	3.2 (2.1 — 4.2)	-1.6 (-2.6 — - 0.6)	20.3 (17.9 — 22.5)	22.0 (17.2 — 26.5)	61.8 (60.3 — 63.2)	71.0 (68.6 — 73.3)	61.6 (59.3 — 63.7)	41.9 (37.2 — 46.3)
Booster with BNT162b2								
0-13	41.6 (40.4 — 42.7)	41.7 (40.5 — 42.9)	39.9 (35.1 — 44.3)	38.9 (23.5 — 51.3)	78.1 (75.1 — 80.7)	84.5 (80.7 — 87.5)	73.9 (68.3 — 78.5)	56.4 (40.0 — 68.3)
14-30	63.6 (62.8 — 64.3)	62.3 (61.5 — 63.1)	71.3 (69.3 — 73.2)	67.7 (60.7 — 73.4)	89.4 (87.8 — 90.7)	91.9 (89.6 — 93.8)	89 (86.7 — 90.9)	79.8 (72.6 — 85.1)
31-60	48.5 (47.8 — 49.3)	44.5 (43.5 — 45.4)	57.2 (55.7 — 58.6)	60.5 (56.3 — 64.3)	89.6 (88.8 — 90.4)	93.1 (91.5 — 94.5)	89.2 (88.2 — 90.2)	81.3 (77.9 — 84.2)
61-90	32.5 (31.7 — 33.3)	30.3 (29.3 — 31.4)	40.3 (38.6 — 41.9)	51.5 (47.9 — 54.9)	89.3 (88.8 — 89.8)	91.5 (90.0 — 92.8)	89.9 (89.2 — 90.5)	77.5 (74.9 — 79.8)
91-120	20.6 (19.7 — 21.5)	19.5 (18.4 — 20.6)	32.0 (30.1 — 33.9)	34.7 (30.6 — 38.4)	87.8 (87.3 — 88.3)	93.3 (92.2 — 94.2)	89.1 (88.4 — 89.8)	74.8 (72.6 — 76.8)
>120	1.7 (0.1 — 3.2)	−1.7 (−4.0 — 0.5)	25.7 (23.1 — 28.2)	30.4 (26.0 — 34.4)	84.1 (83.2 — 84.9)	91.9 (89.4 — 93.7)	86.4 (85.2 — 87.5)	72.9 (70.6 — 75.1)

Supplementary Table 5: Vaccine effectiveness [%-(95% CI)] against death associated with COVID-19 during the Omicron dominance period, stratified by age group

Vaccination Status	Overall	18-59 years	60-79 years	≥ 80 years
First dose				
≥ 14	51.8 (46.5 — 56.5)	52.8 (42.1 —61.5)	53.2 (45.2 —60.1)	42.7 (31.3 —52.2)
Second dose				
14-180	67.8 (64.0 —71.3)	74.5 (70.3 —78.1)	54.8 (43.6 —63.8)	56.1 (42.1 —66.6)
> 180	63.1 (60.9 —65.1)	78.3 (73.9 —81.9)	64.2 (61.1 —67.0)	49.2 (44.1 —53.8)
Booster with BNT162b2				
0-13	84.4 (79.9 — 87.9)	88.2 (79.1 — 93.4)	84.9 (78.3 — 89.5)	75.8 (61.2 — 84.9)
14-30	90.2 (87.6 — 92.3)	97.2 (92.4 — 98.9)	88.3 (84.4 — 91.3)	87.5 (80.3 — 92.1)
31-60	90.5 (89.3 — 91.6)	96.1 (92.9 — 97.9)	90.8 (89.2 — 92.1)	85.3 (81.5 — 88.4)
61-90	90.6 (89.8 — 91.3)	97.0 (94.7 — 98.3)	91.9 (91.0 — 92.7)	80.9 (77.9 — 83.4)
91-120	89.7 (88.9 — 90.3)	95.1 (93.0 — 96.6)	91.4 (90.5 — 92.2)	81.2 (79.1 — 83.1)
>120	87.0 (85.9 — 88.0)	93.8 (88.8 — 96.6)	89.9 (88.4 — 91.2)	80.2 (78.0 — 82.3)

Supplementary Table 6: Adjusted odds ratio (95% CI) comparing odds of a booster dose in cases and controls, reference group individuals past 180 days of the second dose without a booster. Models for symptomatic and severe COVID-19. Odds ratio according to days past booster dose during the Omicron dominance period, stratified by age group.

Symptomatic Infection								
Days since booster dose	Unadjusted Odds Ratio*				Adjusted Odds Ratio‡			
	Overall	18-59 years	60-79 years	≥ 80 years	Overall	18-59 years	60-79 years	≥ 80 years
0-13	0.566 (0.577 — 0.555)	0.55 (0.561 — 0.538)	0.764 (0.822 — 0.711)	0.817 (1.017 — 0.656)	0.591 (0.603 — 0.579)	0.566 (0.578 — 0.554)	0.753 (0.810 — 0.699)	0.784 (0.979 — 0.628)
14-30	0.367 (0.374 — 0.359)	0.369 (0.378 — 0.361)	0.37 (0.395 — 0.348)	0.411 (0.496 — 0.341)	0.371 (0.379 — 0.363)	0.368 (0.377 — 0.360)	0.359 (0.383 — 0.337)	0.412 (0.499 — 0.341)
31-60	0.532 (0.540 — 0.525)	0.543 (0.554 — 0.533)	0.559 (0.574 — 0.544)	0.49 (0.537 — 0.447)	0.53 (0.538 — 0.522)	0.546 (0.557 — 0.536)	0.534 (0.549 — 0.520)	0.502 (0.551 — 0.457)
61-90	0.69 (0.698 — 0.682)	0.653 (0.664 — 0.643)	0.744 (0.757 — 0.730)	0.616 (0.653 — 0.581)	0.697 (0.705 — 0.688)	0.682 (0.693 — 0.671)	0.748 (0.762 — 0.734)	0.619 (0.657 — 0.583)
91-120	0.801 (0.810 — 0.793)	0.767 (0.778 — 0.756)	0.805 (0.819 — 0.790)	0.838 (0.875 — 0.802)	0.823 (0.833 — 0.815)	0.795 (0.806 — 0.783)	0.854 (0.870 — 0.838)	0.836 (0.875 — 0.800)
>120	1.019 (1.035 — 1.004)	0.993 (1.015 — 0.971)	0.872 (0.895 — 0.849)	0.902 (0.944 — 0.862)	1.029 (1.045 — 1.013)	1.015 (1.038 — 0.992)	0.934 (0.960 — 0.908)	0.9 (0.944 — 0.859)
Severe Outcomes								
Days since booster dose	Unadjusted Odds Ratio*				Adjusted Odds Ratio‡			
	Overall	18-59 years	60-79 years	≥ 80 years	Overall	18-59 years	60-79 years	≥ 80 years

0-13	0.111 (0.124 — 0.099)	0.281 (0.350 — 0.225)	0.543 (0.643 — 0.459)	0.767 (1.017 — 0.579)	0.601 (0.683 — 0.528)	0.51 (0.640 — 0.407)	0.679 (0.823 — 0.561)	0.758 (1.039 — 0.552)
14-30	0.084 (0.095 — 0.074)	0.184 (0.238 — 0.142)	0.27 (0.320 — 0.228)	0.341 (0.448 — 0.259)	0.288 (0.329 — 0.251)	0.263 (0.343 — 0.201)	0.285 (0.343 — 0.237)	0.347 (0.470 — 0.257)
31-60	0.188 (0.201 — 0.177)	0.209 (0.260 — 0.169)	0.233 (0.252 — 0.216)	0.301 (0.348 — 0.260)	0.275 (0.296 — 0.256)	0.231 (0.288 — 0.185)	0.277 (0.302 — 0.254)	0.32 (0.376 — 0.272)
61-90	0.308 (0.320 — 0.296)	0.268 (0.319 — 0.226)	0.234 (0.245 — 0.223)	0.356 (0.389 — 0.326)	0.28 (0.293 — 0.267)	0.289 (0.345 — 0.241)	0.261 (0.276 — 0.247)	0.386 (0.426 — 0.350)
91-120	0.391 (0.404 — 0.378)	0.224 (0.261 — 0.192)	0.286 (0.299 — 0.274)	0.407 (0.432 — 0.382)	0.314 (0.326 — 0.302)	0.229 (0.269 — 0.195)	0.282 (0.297 — 0.268)	0.436 (0.467 — 0.407)
>120	1.021 (1.063 — 0.982)	0.311 (0.403 — 0.240)	0.399 (0.426 — 0.373)	0.489 (0.520 — 0.459)	0.402 (0.422 — 0.382)	0.28 (0.366 — 0.214)	0.354 (0.382 — 0.327)	0.472 (0.508 — 0.439)

* Unadjusted odds ratio= model including the vaccination status term adjusted only for temporal trend (penalized cubic spline)

‡ Adjusted odds ratio= model including vaccination status adjusted for: temporal trend (penalized cubic spline), age (penalized cubic spline), previous infection, sex, geography (state of residence, residence in state capital and deprivation quintile of the city), comorbidities (*Diabetes Mellitus*, obesity, immunosuppression, chronic respiratory disease, chronic cardiac disease, chronic kidney disease).

Supplementary Table 7: Vaccine effectiveness [%-(95% CI)] against symptomatic SARS-CoV-2 infection during the Omicron dominance period, stratified by type of test and age group.

Days since booster dose	RT-PCR				Lateral-flow			
	Overall	18-59 years	60-79 years	≥ 80 years	Overall	18-59 years	60-79 years	≥ 80 years
0-13	30.6 (27.0 — 34.0)	29.4 (25.5 — 33.0)	36.4 (22.9 — 47.6)	32.7 (−8.5 — 58.3)	42.6 (41.3 — 43.8)	43.1 (41.8 — 44.3)	38.4 (33.0 — 43.4)	39.6 (21.7 — 53.4)
14-30	61.3 (59.3 — 63.1)	58.1 (55.8 — 60.2)	74.4 (70.1 — 78.0)	66.2 (51.6 — 76.4)	64.0 (63.2 — 64.8)	63.2 (62.3 — 64.1)	69.8 (67.4 — 72.0)	67.8 (59.3 — 74.5)
31-60	50.8 (49.0 — 52.6)	43.4 (40.8 — 45.9)	59.9 (56.8 — 62.8)	65.5 (58.4 — 71.3)	48.0 (47.2 — 48.9)	44.8 (43.7 — 45.9)	55.6 (53.8 — 57.2)	57.9 (52.4 — 62.7)
61-90	36.5 (34.6 — 38.3)	31.8 (29.3 — 34.3)	44.2 (40.6 — 47.6)	58.5 (52.7 — 63.7)	32.5 (31.6 — 33.4)	32.3 (31.1 — 33.4)	37.7 (35.7 — 39.6)	47.2 (42.4 — 51.6)
91-120	27.9 (26.0 — 29.9)	29.2 (26.9 — 31.5)	34.1 (29.8 — 38.2)	39.0 (32.0 — 45.3)	20.6 (19.6 — 21.5)	20.0 (18.8 — 21.2)	29.6 (27.3 — 31.7)	30.9 (25.7 — 35.8)
>120	15.2 (11.7 — 18.6)	11.6 (6.3 — 16.6)	36.0 (30.4 — 41.1)	36.7 (29.0 — 43.5)	1.5 (−0.2 — 3.2)	−2.6 (−5.0 — −0.2)	22.4 (19.4 — 25.3)	25.8 (20.2 — 31.0)

Supplementary Table 8: Vaccine effectiveness against [%-(95% CI)] severe outcomes associated with COVID-19 during the Omicron dominance period, stratified by type of test and age group.

Days since booster dose	RT-PCR				Lateral-flow			
	Overall	18-59 years	60-79 years	≥ 80 years	Overall	18-59 years	60-79 years	≥ 80 years
0-13	73.4 (67.9 — 77.9)	79.7 (72.9 — 84.8)	66.3 (54.3 — 75.2)	38.3 (−3.6 — 63.3)	77.4 (72.8 — 81.2)	85.0 (79.1 — 89.3)	72.8 (64.4 — 79.3)	63.2 (42.3 — 76.5)
14-30	88.3 (85.8 — 90.3)	91 (87.0 — 93.7)	87.3 (83.3 — 90.3)	69.9 (53.5 — 80.5)	88.9 (86.5 — 90.9)	91.1 (87.1 — 93.8)	88.6 (85.0 — 91.3)	84.9 (76.0 — 90.5)
31-60	88 (86.7 — 89.2)	91.7 (89.1 — 93.7)	86.3 (84.3 — 88.0)	76.0 (69.7 — 81.0)	89.8 (88.6 — 90.9)	93.9 (91.4 — 95.7)	89.7 (88.1 — 91.0)	84.3 (79.8 — 87.8)
61-90	86.8 (85.8 — 87.7)	91.2 (89.0 — 92.9)	85.6 (84.1 — 87.0)	72.2 (67.4 — 76.3)	89.8 (89.0 — 90.5)	92.2 (89.9 — 94.1)	90.7 (89.8 — 91.6)	79.4 (75.8 — 82.4)
91-120	83.5 (82.5 — 84.6)	93.2 (91.8 — 94.4)	82.6 (80.8 — 84.2)	62.5 (57.5 — 66.9)	89.2 (88.5 — 89.9)	94.2 (92.6 — 95.4)	90.7 (89.8 — 91.5)	80.3 (77.7 — 82.5)
>120	81.1 (79.6 — 82.6)	92.3 (88.9 — 94.7)	80.7 (78.1 — 83.0)	62.3 (57.0 — 66.8)	85.0 (83.8 — 86.1)	91.4 (87.6 — 94.1)	87.3 (85.7 — 88.8)	77.1 (74.2 — 79.7)

Supplementary Figure 1: Monthly prevalence of SARS-CoV-2 variants in Brazil among genotyped isolates in the GISAID (global initiative on sharing avian influenza data).

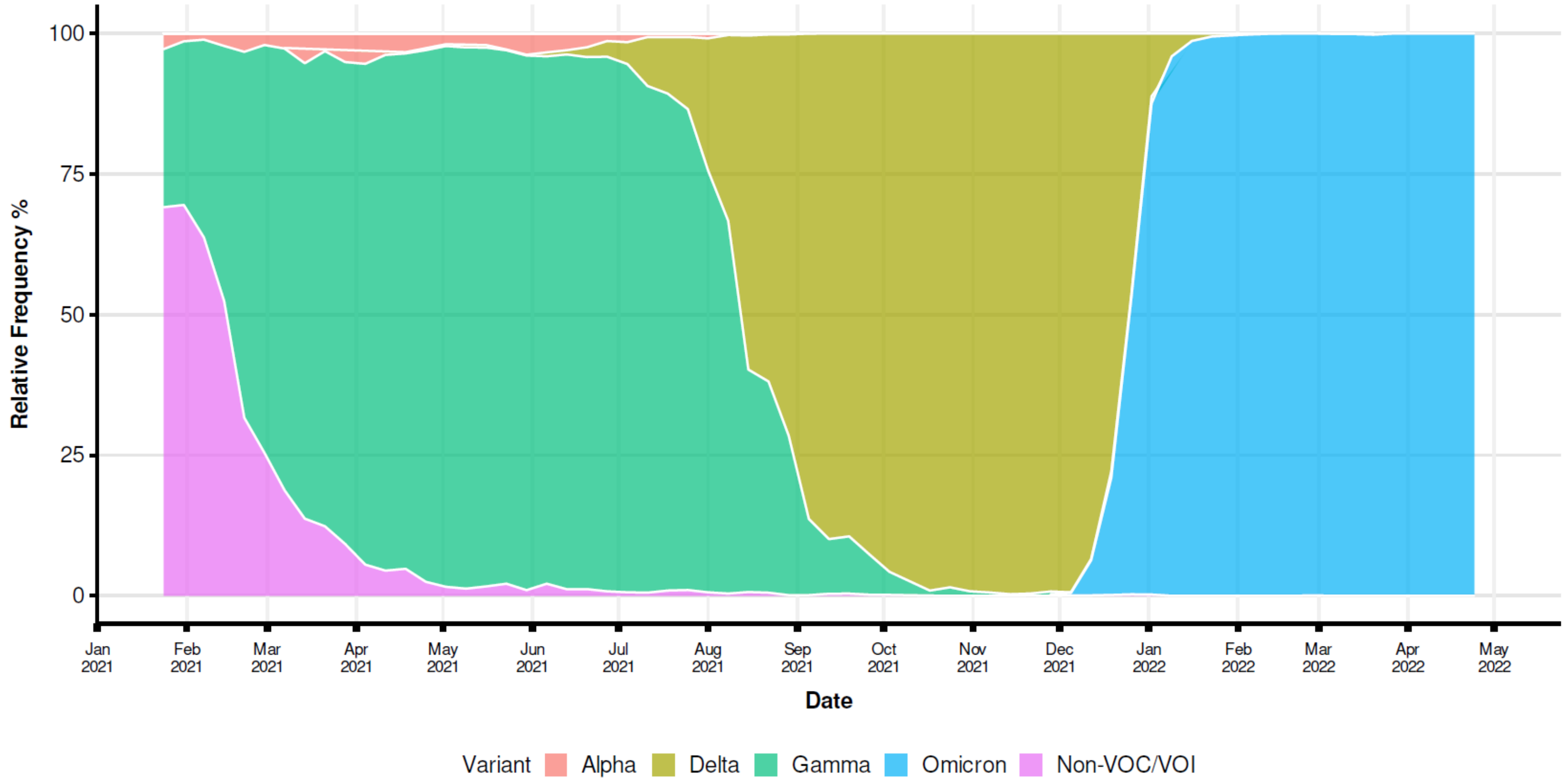

Supplementary Figure 2: Adjusted odds ratio comparing odds of a booster dose in cases and controls. Models for symptomatic and severe COVID-19. Odds ratio according to days post booster dose during the Omicron dominance period, stratified by age group. Circles are adjusted odds ratio, with error bars indicating the corresponding 95% Wald's C.I.

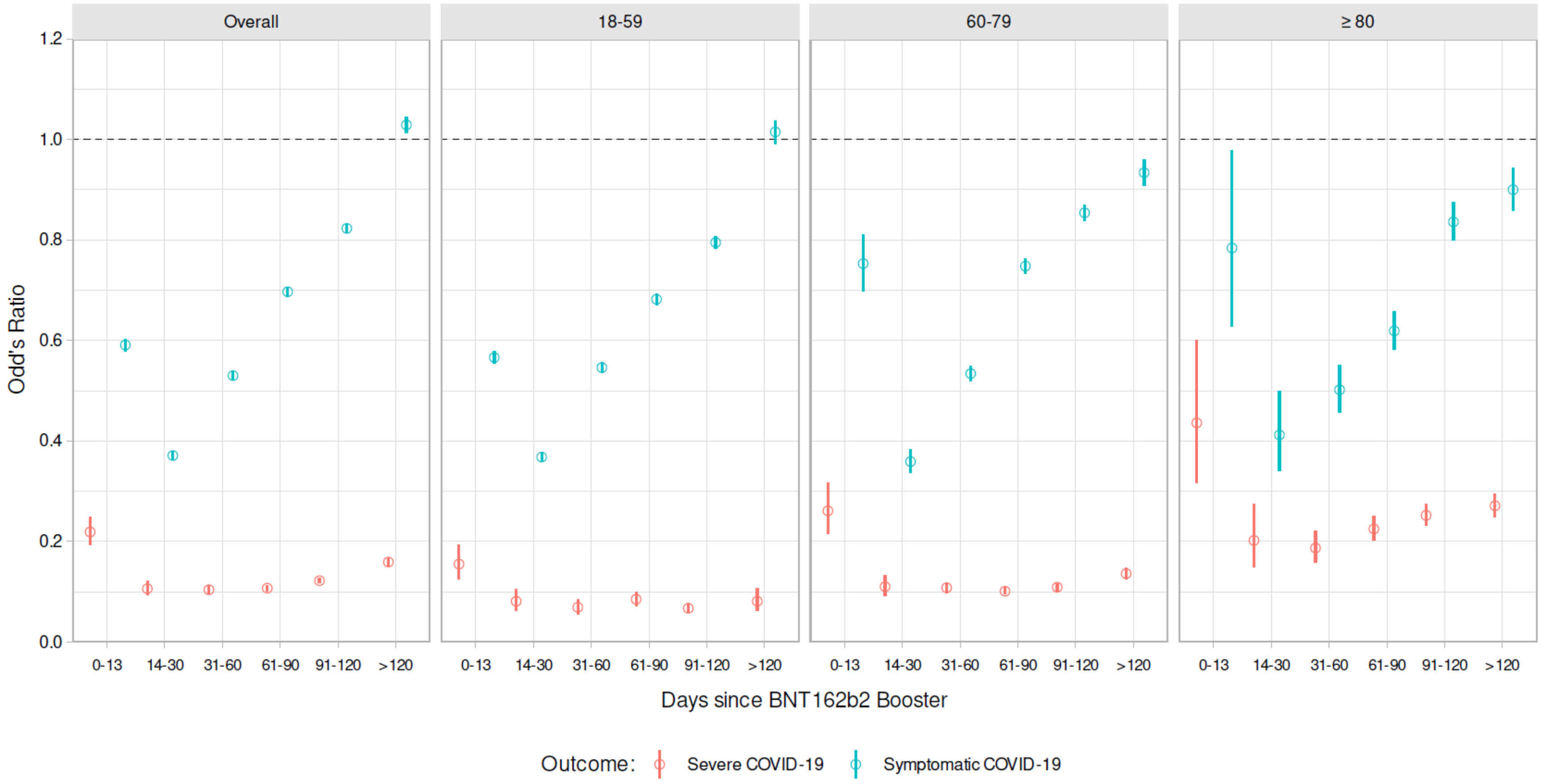

Adjusted odds ratio= model including vaccination status adjusted for: temporal trend (penalized cubic spline), age (penalized cubic spline), previous infection, sex, geography (state of residence, residence in state capital and deprivation quintile of the city), comorbidities (*Diabetes Mellitus*, obesity, immunosuppression, chronic respiratory disease, chronic cardiac disease, chronic kidney disease).

Supplementary figure 3- Diagram showing the data linkage process

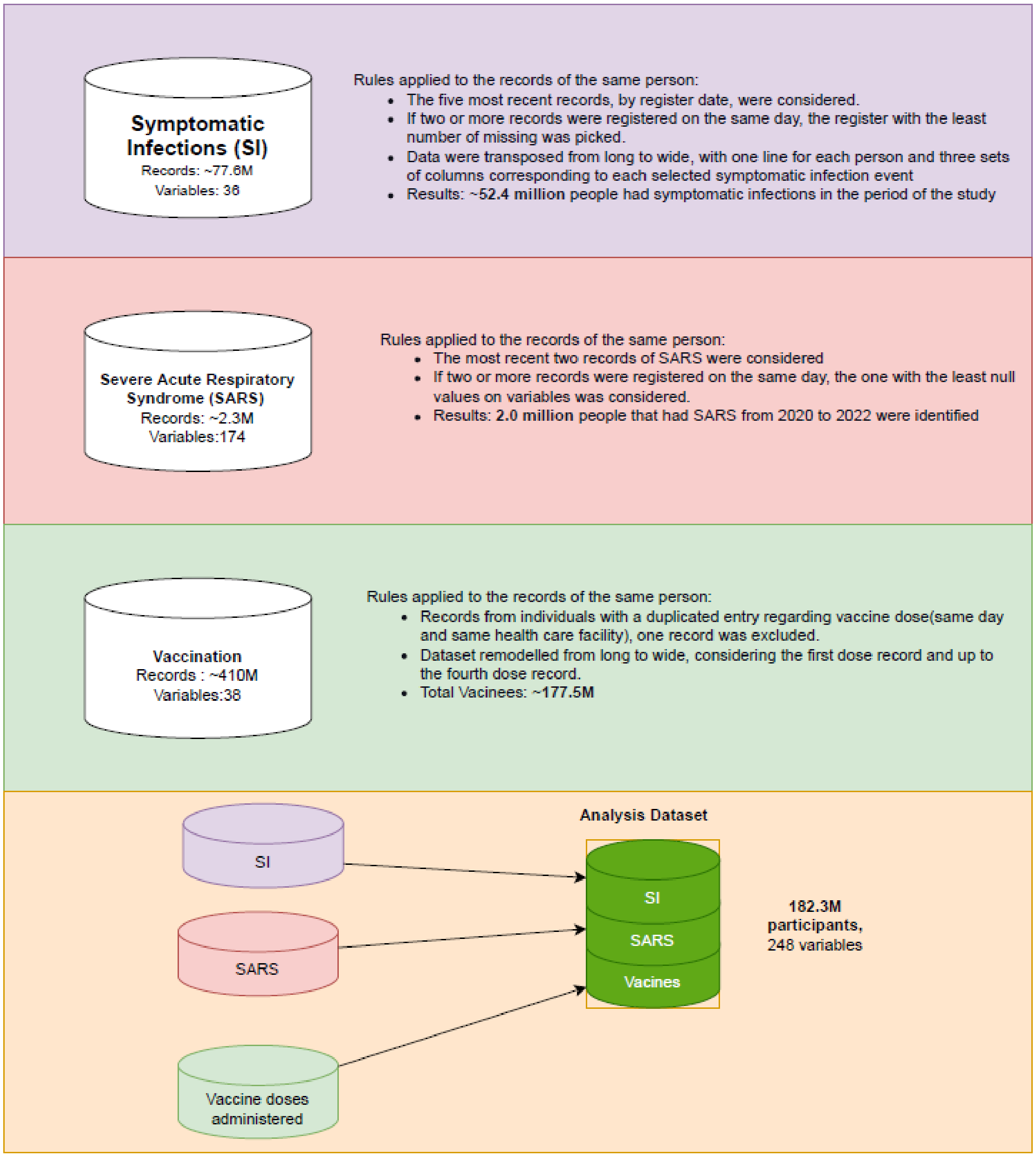

The RECORD statement – checklist of items, extended from the STROBE statement, that should be reported in observational studies using routinely collected health data.

	Item No.	STROBE items	RECORD items	Location in manuscript where items are reported
**Title and abstract**				
	1	(a) Indicate the study's design with a commonly used term in the title or the abstract (b) Provide in the abstract an informative and balanced summary of what was done and what was found	RECORD 1.1: The type of data used should be specified in the title or abstract. When possible, the name of the databases used should be included.<br>RECORD 1.2: If applicable, the geographic region and timeframe within which the study took place should be reported in the title or abstract.<br>RECORD 1.3: If linkage between databases was conducted for the study, this should be clearly stated in the title or abstract.	1.1. The type of data is described, and details in the Methods second paragraph.<br>1.2. Time and region described in the Abstract and methods<br>1.3. Linkage of databases pointed in the methods third paragraph
**Introduction**				

Background rationale	2	Explain the scientific<br>background and rationale for the investigation being reported		Main Section, paragraphs 1 and 2
Objectives	3	State specific objectives, including any prespecified hypotheses		Main Section, paragraph 3
**Methods**				
Study Design	4	Present key elements of study design early in the paper		Methods paragraph 1
Setting	5	Describe the setting, locations, and relevant dates, including periods of recruitment, exposure, follow-up, and data collection		Methods paragraph 2,3,4
Participants	6	*(a)* Cohort study - Give the eligibility criteria, and the sources and methods of selection of participants. Describe methods of follow-up<br>Case-control study - Give the eligibility criteria, and the sources and methods of case ascertainment and control selection. Give the rationale for the choice of cases and controls<br>Cross-sectional study - Give the eligibility criteria, and the	RECORD 6.1: The methods of study population selection (such as codes or algorithms used to identify subjects) should be listed in detail. If this is not possible, an explanation should be provided.<br><br>RECORD 6.2: Any validation studies of the codes or algorithms used to select the population should be referenced. If validation was conducted for this study and not	6.1 – Information was included in:<br>- data sources<br>- Study design, population, and data sources (paragraph 3 of Methods section)<br>-<br><br>6.2.NA

		sources and methods of selection of participants<br><br>*(b)* Cohort study - For matched studies, give matching criteria and number of exposed and unexposed<br>Case-control study - For matched studies, give matching criteria and the number of controls per case	published elsewhere, detailed methods and results should be provided.<br><br>RECORD 6.3: If the study involved the linkage of databases, consider use of a flow diagram or other graphical display to demonstrate the data linkage process, including the number of individuals with linked data at each stage.	6.3. Supplementary Figure 4- Data Linkage Diagram
Variables	7	Clearly define all outcomes, exposures, predictors, potential confounders, and effect modifiers. Give diagnostic criteria, if applicable.	RECORD 7.1: A complete list of codes and algorithms used to classify exposures, outcomes, confounders, and effect modifiers should be provided. If these cannot be reported, an explanation should be provided.	Methods paragraph 4
Data sources/ measurement	8	For each variable of interest, give sources of data and details of methods of assessment (measurement).<br><br>Describe comparability of assessment methods if there is<br><br>more than one group		Methods -Statistical analyses section
Bias	9	Describe any efforts to address potential sources of bias		Discussion paragraph 4

Study size	10	Explain how the study size was arrived at		We included all Brazilian individuals who have tested for SARS-CoV-2, This information was included in Study design, population, and data sources section, and in the Flowchart (supplementary material)
Quantitative variables	11	Explain how quantitative variables were handled in the analyses. If applicable, describe which groupings were chosen, and why		Described in Statistical analyses section
Statistical methods	12	(a) Describe all statistical methods, including those used to control for confounding (b) Describe any methods used to examine subgroups and interactions<br>(c) Explain how missing data were addressed<br>(d) Cohort study - If applicable, explain how loss to follow-up was addressed<br>Case-control study - If applicable, explain how matching of cases and controls was addressed<br>Cross-sectional study - If applicable, describe analytical methods taking account of sampling strategy		(a) Described in Statistical analyses section<br>(b) Described Study design, population, and data sources and in the Statistical analyses section<br>(c) Described at the end of Statistical Analyses section<br>(d) Not applicable to the study design<br>(e) Described in the Statistical analyses section

		(e) Describe any sensitivity analyses		
Data access and cleaning methods			RECORD 12.1: Authors should describe the extent to which the investigators had access to the database population used to create the study population.	As described in Study design, population, and data sources, we had access to whole Brazilian population data
			RECORD 12.2: Authors should provide information on the data cleaning methods used in the study.	Flowcharts (Linkage and Study), provided as Supplementary Figures
Linkage			RECORD 12.3: State whether the study included person-level,<br><br>institutional-level, or other data linkage across two or more databases. The methods of linkage and methods of linkage quality evaluation should be provided.	Linkage Flowchart provided in supplementary documents
Participants	13	(a) Report the numbers of individuals at each stage of the study (e.g., numbers potentially eligible, examined for eligibility, confirmed eligible, included in the study, completing follow-up, and analysed)<br>(b) Give reasons for nonparticipation at each	RECORD 13.1: Describe in detail the selection of the persons included in the study (i.e., study population selection) including filtering based on data quality, data availability and linkage. The selection of included persons can be described in the text and/or by means of the study flow diagram.	Flowcharts are provided for the Linkage and Study population selection

		stage. (c) Consider use of a flow diagram		
Descriptive data	14	(a) Give characteristics of study participants (e.g., demographic, clinical, social) and information on exposures and potential confounders<br>(b) Indicate the number of participants with missing data for each variable of interest (c) Cohort study - summarise follow-up time (e.g., average and total amount)		In the Results section
Outcome data	15	Cohort study - Report numbers of outcome events or summary measures over time<br><br>Case-control study - Report numbers in each exposure category, or summary measures of exposure<br><br>Cross-sectional study - Report numbers of outcome events or summary measures		Provided as a Table 1 and Supplementary Table 1
Main results	16	(a) Give unadjusted estimates and, if applicable, confounderadjusted estimates and their precision (e.g., 95% confidence interval). Make clear which confounders were		In the Results Section

		adjusted for and why they were included (b) Report category boundaries when continuous variables were categorized<br>(c) If relevant, consider translating estimates of relative risk into absolute risk for a meaningful time period		
Other analyses	17	Report other analyses done—<br>e.g., analyses of subgroups and interactions, and sensitivity analyses		Sensitivity and Subgroups analyses are presented in the Discussion and Statistical analysis section
Key results	18	Summarise key results with reference to study objectives		Summarised both in abstracts and results
Limitations	19	Discuss limitations of the study, taking into account sources of potential bias or imprecision. Discuss both direction and magnitude of any potential bias	RECORD 19.1: Discuss the<br>implications of using data that were not created or collected to answer the specific research question(s). Include discussion of misclassification bias, unmeasured confounding, missing data, and changing eligibility over time, as they pertain to the study being reported.	In discussion section
Interpretation	20	Give a cautious overall interpretation of results considering objectives,		Discussion

		limitations, multiplicity of analyses, results from similar studies, and other relevant evidence		Discussion
Generalisability	21	Discuss the generalisability (external validity) of the study results		Discussion
Funding	22	Give the source of funding and the role of the funders for the present study and, if applicable, for the original study on which the present article is based		Acknowledgements
Accessibility of protocol, raw data, and programming code			RECORD 22.1: Authors should provide information on how to access any supplemental information such as the study protocol, raw data, or programming code.	This information are available at: https://vigivac.fiocruz.br – except for raw data, which is protected under Brazilian personal data protection law (LGPD), as explained in the Data availability section
